# The Activity of Superoxide Dismutase, Its Relationship with the Concentration of Zinc and Copper and the Prevalence of rs2070424 Superoxide Dismutase Gene in Women with Polycystic Ovary Syndrome—Preliminary Study

**DOI:** 10.3390/jcm11092548

**Published:** 2022-05-01

**Authors:** Anna Bizoń, Agata Tchórz, Paweł Madej, Marcin Leśniewski, Mariusz Wójtowicz, Agnieszka Piwowar, Grzegorz Franik

**Affiliations:** 1 Department of Toxicology/Department of Biomedical and Environmental Analyses, Faculty of Pharmacy, Wroclaw Medical University, 50-556 Wrocław, Poland; 2 Students Scientific Society at the Department of Biomedical and Environmental Analyses, Faculty of Pharmacy, Wroclaw Medical University, 50-556 Wrocław, Poland; agata.tchorz@student.umw.edu.pl; 3 Department of Endocrinological Gynecology, Medical University of Silesia, 40-752 Katowice, Poland; pmadej@sum.edu.pl (P.M.); franik@sum.edu.pl (G.F.); 4 Department of Gynaecological and Obstetrics, District Hospital, 43-200 Pszczyna, Poland; marcinlesniewski@wp.pl; 5 Women’s and Child Health Center, City Hospital, 41-803 Zabrze, Poland; mariuszwojtowicz007@onet.eu; 6 Department of Toxicology, Faculty of Pharmacy, Wroclaw Medical University, 50-556 Wrocław, Poland; agnieszka.piwowar@umw.edu.pl

**Keywords:** polycystic ovary syndrome, superoxide dismutase, trace elements

## Abstract

Superoxide dismutase (SOD) is a crucial antioxidant enzyme involved in the antioxidant pathway during both normal cellular metabolism and different pathologic processes. We investigated the activity of the copper (Cu)-zinc (Zn) SOD1 as well as the level of Cu and Zn in the serum of women with polycystic ovary syndrome (PCOS) and control group. Moreover, the prevalence of rs2070424 gene polymorphism of the enzyme in the course of PCOS was evaluated. Significantly lower activity of SOD 1 and Cu, Zn concentration was found in the group of women with PCOS than without the syndrome. Insulin resistance in the group of women with PCOS caused a further SOD1 activity decrease, while Cu concentration and the value of Cu/Zn was increased when compared to women with normal insulin levels. Furthermore, we assessed for the first time the rs2070424 polymorphism of SOD1 in the women with PCOS, and in these patients we detected dominant variant AA (93.3%). Due to a small number of other genotypes, it is difficult to state if lower SOD1 activity was strictly associated with the AA variant or if other factors play a crucial role, but this should be taken into account.

## 1. Introduction

Polycystic ovary syndrome (PCOS) is associated with multiple disorders, not only in areas of metabolism or hormones, but also with social and psychological disturbances, which have impacts on women’s health across their lifespans [1]. Many of these disturbances could be related to oxidative stress and altered homeostasis of essential elements [2]. If reactive oxygen species (ROS) production is maintained at high levels, the system’s defenses against ROS can be overwhelmed, which may lead to disease conditions [3].

One of the most important antioxidant enzymes is superoxide dismutase (SOD), which converts the anion superoxide (O_2_^−^) into hydrogen peroxide (H_2_O_2_) [4]. This reaction is a primary step in the antioxidant pathway during both normal cellular metabolism and different pathologic processes [5]. All mammalian cells possess three isoforms of superoxide dismutase enzymes; the cytosolic copper (Cu)-zinc (Zn) dimeric form, (SOD1; Cu/Zn SOD), the mitochondrial tetrameric manganese (SOD2; MnSOD) and the extracellular tetrameric (SOD3; EC-SOD). The activity of SOD1 accounts for 50–80% of total SOD activity [6], therefore changes in SOD1 activity are the most important.

The alterations in the expression level, concentration and activity of SOD were revealed in diverse pathological conditions such as cancer, cardiovascular, neurodegenerative and metabolic diseases. It is noteworthy that the changes in SOD status could be associated with both the risk of developing disorders, as well as with their course and exacerbation [7,8].

In case of women’s genital functions, SOD activity was identified in developing follicles, membranes of granulosa cells of Graafian follicles, postovulatory follicles as well as in follicular fluid and in ovaries, which indicates that this enzyme is involved in the ovulation process and the development of oocytes [5]. It addition, it was shown that estrogen and progestin therapy in postmenopausal women increased total antioxidant capacity including SOD activity [9] and that the antioxidant effect of estrogen may be associated with a direct free radical scavenging potential [10], whereas the influence of androgen on SOD activity is inconsistent. One study has shown that androgens such as dihydrotestosterone could induce SOD activity to enhance resistance to oxidative stress [11], while another revealed that higher testosterone concentration can depress the activity of SOD [12]. Furthermore, SOD mimetics were proposed as a novel class of androgen receptor inhibitors that suppress growth of prostate cancer [13].

The variability in SOD activity and the harmful consequences of oxidative stress may be also related to intra-individual differences, presumed to be associated with single-nucleotide polymorphisms (SNP) of SOD1. One of them could be rs2070424 (A251G) of the SOD1 gene, which is located in the 3rd intron of the SOD1 gene [14]. This polymorphism could be associated with increased body fat [15], impaired glucose regulation, type 2 diabetes [16] and inflammation [17], which are common disorders observed in the course of PCOS. In the Chinese Han population, it was found that the risk of newly diagnosed type 2 diabetes or impaired glucose regulation was associated with the prevalence of allele A of rs2070424 [16]. Furthermore, Haldar et al. evaluated the associations between the single-nucleotide polymorphisms of important antioxidant parameters with presence of diabetes and they revealed that the prevalence of allele A of rs2070424 was one of the significant factors affecting occurrence of diabetes [18].

Moreover, SOD1 activity is closely associated with Cu and Zn concentrations [4]. In human SOD1, catalysis occurs at the Cu site, whereas the Zn site is important for the support of the protein structure. Cu is involved in immune functions, energy production and glucose metabolism, whereas excess copper concentration has been revealed to accompany higher ROS levels and increased insulin resistance [16]. Zn plays essential role in cell signaling, enzymatic activities, regulation of normal growth and puberty process, or mitochondrial oxidative stress [19]. Furthermore, both Cu and Zn are essential for reproductive health [20].

Therefore, in the present study, we aimed to evaluate the activity of SOD1 and the concentrations of Cu and Zn in a group of women with and without PCOS. Moreover, we evaluated whether the alteration of the activity of SOD1 and the concentrations of Cu and Zn could be associated with polymorphism of SOD1, especially rs2070424, which could be crucial in pathogenesis and course of PCOS.

## 2. Materials and Methods

The study included 75 women: *n* = 60 with PCOS and *n* = 15 women without PCOS or any other diseases, of a similar age and BMI-matched. Diagnosis of PCOS was conducted in the Gynecological Endocrinology Clinic of the Medical University of Silesia in Katowice, Poland according to the Rotterdam ESHRE/ASRM criteria from 2003 [21] with at least two out of the three following criteria: the existence of oligomenorrhea or amenorrhea, clinical or biochemical hyperandrogenism, and ovarian polycystic morphology in ultrasonography. Hypertension, Cushing’s syndrome, androgen-secreting tumor, thyroid dysfunctions or type 1 and 2 diabetes were exclusion criteria in the group of women with PCOS. In addition, in the group of women without PCOS, cardiovascular diseases, liver diseases, diabetes, hypertension, inflammation, cancer or other infections were excluded. In both groups, women who had taken any drugs or supplements in the prior 6 months were also excluded from the study. Alcohol abuse and cigarette smoking were also among the exclusion criteria.

The blood samples were collected into two different tubes after overnight fasting. The first portion of whole blood collected on EDTA was used for DNA isolation, and from the second portion, serum was obtained for determination of SOD1 activity, the concentrations of Cu and Zn.

Moreover, in the group of women with PCOS, the concentrations of lipid profile parameters (total cholesterol, high-density-lipoprotein cholesterol—HDL-C, low-density lipoprotein cholesterol—LDL-C and triglycerides), glucose and insulin (both fasting and after oral glucose tolerance test) and hormones (luteinizing hormone (LH); follicle-stimulating hormone (FSH); free thyroxine (FT4); thyrotropin (TSH); sex hormone-binding globulin (SHBG); 17 β-estradiol (17-β-E2); 17-α-hydroxyprogesterone (17-OHP), free and total testosterone; androstenedione) were measured during routine diagnostic process [22]. In contrast, in the control group, only fasting concentrations of lipid profile parameters, glucose and insulin were assayed.

The activity of SOD in plasma was determined with using a commercial kit (Cat. No.: 706002, Cayman Chemical, Ann Arbor, MI, USA). The serum concentrations of Cu and Zn were assayed using flame atomic absorption spectrometry (FAAS) in an air-acetylene flame on Solaar M6 (Solaar House, Cambridge, UK) in the Atomic Absorption Spectroscopy Laboratory of the Department and Clinic of Internal and Occupational Diseases, Wroclaw Medical University in Poland. The repeatability and accuracy of the method was verified using the concentration of Cu and Cu in control serum samples (Seronorm Trace Elements; Sero AS, Billingstad, Norway, reference No. 201405) with known concentrations of the parameters (Cu, 1.19 mg/L; Zn, 1.30 mg/L).

The evaluation of SNP rs2070424 in the SOD1 gene was performed using the polymerase chain reaction–restriction fragment length polymorphism (PCR–RFLP) procedure. Firstly, DNA was isolated from the whole blood collected on EDTA with use of Syngen Blood/Cell DNA Mini Kit (Syngen Biotech, Cat. No: SY221012, Wrocław, Poland). The PCR reaction was performed with 2 μL of isolated DNA, 4 μL Gold Hot Start PCR Mix (Syngen Biotech, Cat. No.: SY550231, Poland), 0.6 μL of each primer (forward: AGTACTGTCAACCACTAGCA and reverse: CCAGTGTGCGGCCAATGATG) and 12.8 μL PCR clean water (Syngen Biotech, Cat. No.: SY550231, Poland) under the following conditions: the initial denaturation at 95 °C for 15 min, followed by 35 cycles of: denaturation at 95 °C for 40 s, annealing at 60 °C for 35 s, elongation at 72 °C for 45 s, and a final elongation step at 72 °C for 15 min. The PCR-RFLP products were received with MspI (10 U/μL) restriction enzyme (Thermo Fischer, Cat. No.: ER0541, Waltham, MA, USA), and the digested fragments were visualized in 3% agarose gel (Syngen Biotech, Cat. No.: SY521011, Poland) with dye GreenDNA Gel Stain (Syngen Biotech, Cat. No.: SY521032, Poland) and GeneRuler Ultra Low Range DNA Ladder (Thermo Fisher Scientific, Cat. No.: SM1213, USA). Digested DNA fragments of 369 and 201 bp were detected in GG homozygotes (restriction site present); an undigested band of 570 bp was detected in AA homozygotes (restriction site absent); and AG heterozygous genotypes resulted in three different bands (570, 369, 201 bp).

### Statistical Analysis

The statistical analysis was performed using the Statistica software package, version 13.3. The normality of distribution was tested by the Shapiro–Wilk W-test, while the homogeneity of variance was determined using Levene’s test. Due to a lack of normal distribution, a non-parametric Mann–Whitney U test was used. The values were expressed as median and 1st quartile, and 3rd quartile. The correlation was checked using Spearman’s rank-order correlation coefficient. In all cases, a *p* value lower than 0.05 was considered statistically significant.

## 3. Results

The basic anthropometric and biochemical characteristics and examined parameters (SOD1 activity, Cu and Zn concentration, Cu/Zn ratio) of the women with and without PCOS were summarized in Table 1. There were no statistically significant differences regarding the age, BMI and WHR between both groups. We did not observe any significant differences in glucose metabolism parameters and lipid profile parameters between women with and without PCOS. The women with PCOS were characterized by significantly lower serum Zn and Cu concentrations and the value of the Cu/Zn ratio when compared to women without PCOS. In addition, decreased SOD1 activity was detected in the group with PCOS.

We also evaluated the activity of SOD and the concentration of Cu and Zn in the subgroups of women with PCOS, dividing them according to occurrence of insulin resistance (HOMA-IR ≥ 2.0) and insulin sensitivity (HOMA-IR < 2.0), as well as their weight, normal weight (BMI < 25.0) and overweight (BMI ≥ 25.0). The subgroup of women with PCOS and a HOMA-IR value ≥2.0 had even lower activity of SOD, but higher concentrations of Cu and values of Cu/Zn ratio than women with PCOS and insulin sensitivity (HOMA-IR < 2.0). BMI did not influence the activity of SOD1, the concentration of Cu, Zn or the value of Cu/Zn ratio in the group of women with PCOS with normal (BMI < 25.0) and higher (BMI ≥ 25.0) value of BMI. Moreover, we analyzed the concentration of selected hormones and SHBG in the divided subgroups according to the value of HOMA-IR and BMI. Higher values of HOMA-IR and BMI were significantly associated with decreased concentrations of SHBG and increased levels of total testosterone, androstenedione and 17-OHP. Moreover, in the subgroup of women with higher BMI, increased concentrations of free testosterone were found when compared to the subgroup of women with normal BMI (Table 2).

The results of examined parameters depending on the *rs2070424* genotypes of SOD1 in PCOS women are summarized in Table 3. Due to a small number of cases in genotype *AG* and the lack of genotype *GG*, we could not be able to perform any statistical analysis.

We found a negative correlation between the activity of SOD1 and BMI or WHR value. In addition, activity of SOD1 was negatively associated with concentrations of glucose and insulin after OGTT, as well as, with concentrations of triglycerides and LH. Furthermore, concentration of Cu was positively associated with the values of BMI, WHR and HOMA-IR, as well as with concentrations of insulin (both fasting and after OGTT), triglycerides and LH. In the case of Zn concentrations, significant correlation was detected only with fasting glucose concentration. Any other statistically significant correlations were not found. The results are presented in Table 4.

## 4. Discussion

The role of oxidative stress in the course of PCOS has been reported [23,24], and is mainly associated with the development of insulin resistance and hyperandrogenism [25]. Previous studies confirmed increased oxidative stress in women with PCOS, but many different types of alterations in pro/antioxidant disorders were observed. For instance, activity of SOD1 in serum of women with PCOS is controversial. The activity of SOD1 in women with PCOS has been generally examined in Asian populations [24,26,27,28].

Similarly to our results, a significant decrease in the activity of SOD1 in serum of women with PCOS when compared to the control group was noticed by other authors [2,11], which was explained by utilization of SOD1 in response to augmented production of ROS due to both hyperglycemia and excess free fatty acids. Moreover, Seleem et al. even proposed that the assay of serum SOD1 activity could be a useful clinical parameter for determining systemic oxidative stress in PCOS patients undergoing intracytoplasmic sperm injection [28]. However, there are also studies demonstrating an elevation in SOD1 activity, which was explained by compensatory response by defense mechanisms to higher oxidative stress in these women [26,27,29].

In our study, even young women with PCOS (median value of age was 24.0) had almost twofold lower activity of SOD1 than in serum of women without PCOS and of a similar age. Moreover, the lowest serum activity of SOD1 was detected in the women with PCOS with IR (the value of HOMA-IR ≥2.0) when compared to subgroups with HOMA-IR <2.0. Lower SOD1 activity was also associated with higher concentrations of glucose and insulin after OGTT, which confirmed that disturbances in glucose metabolism in the course of PCOS could be associated with decreased SOD1 activity. Our results are consistent with earlier investigation conducted by Özer et al., who reported that a drop in SOD1 activity was associated with insulin resistance in women with PCOS [30].

The authors claimed that insulin resistance is a causal factor of the increased oxidative stress in affected women. Moreover, insulin resistance induces hyperglycemia which triggers the release of reactive oxygen species from the mononuclear cells and causes further oxidative stress.

Therefore, it seems that decreased SOD1 activity in the course of PCOS could be associated with disorders of glucose metabolism. We suggest, similarly to Seleem et al. [28], that assays of SOD activity can be useful parameters for monitoring of metabolic disorders (especially glucose metabolism) in the course of PCOS. Moreover, we suggest performing OGTT using a 75 g load not only during PCOS diagnosis, but also in regular follow-ups of women in the course of this disease.

Furthermore, the study conducted by Bellanti et al. reported that estrogens may also affect circulating redox balance by regulating activity of the antioxidative enzymes such as superoxide dismutase, catalase, glutathione peroxidase or glutathione S-transferase [10]. Both in vitro and in vivo studies revealed that 17 β-estradiol can play a crucial role in protection against oxidative damage [10]. Unfortunately, we did not find any significant correlations between SOD1 activity and 17 β -estradiol. When we analyzed the association with other hormones, we revealed only a significant negative correlation between activity of SOD1 and the concentration of LH. Earlier studies conducted on experimental animals revealed that the association between LH concentration and SOD1 activity is dependent on the menstrual cycle phase. LH can increase activity of SOD in the luteal phases and the highest expression of antioxidant enzymes was detected in the midluteal phase. Our study was conducted in the follicular phase of women with PCOS and the activity of SOD1 was presumably decreased by the disturbances observed in the course of PCOS; therefore, we noticed the negative relation between SOD1 activity and LH concentration [8].

Alteration in SOD1 activity might be directly associated not only with metabolic and hormonal disorders, but also with changes in Cu and Zn homeostasis, which are both important elements in the structure and the full enzymatic activity of SOD1 [16]. Similarly to decreased activity of SOD1, we found lower concentrations of Zn and Cu in the serum of women with PCOS when compared to healthy women.

The concentrations of serum Zn and Cu in women with PCOS remain ambiguous. Some investigations show higher, whereas other lower concentrations of Zn in serum of women with PCOS. There are also studies reporting that serum zinc concentration is similar in serum of women with and without PCOS [28,29]. Zn protects the sulfhydryl groups of proteins against oxidation and its deficiency increased oxidative damage in various human cells. Decreased antioxidant capacity can be linked to Zn deficiency and insulin resistance in women with PCOS [30,31,32]. On the other hand, Zn concentration could be modulated by insulin resistance, which could be related to disorders in glucose metabolism parameters in the course of PCOS [33]. Decreased Zn concentrations could be also associated with chronic low-grade inflammation [34], which is also observed during PCOS, especially in obese patients [35]. Unfortunately, we did not observe any significant correlation between the concentration of Zn and the value of HOMA-IR or BMI, which is probably associated with the low BMI value (median 22.7) and young age (median value 24.0) of women with PCOS.

In the case of Cu concentrations, higher [32,36], as well as lower Cu concentration in serum of women with PCOS was also demonstrated when compared to healthy controls [37]. Previous studies show increased Cu levels not only in serum, but also in the follicular fluid, which was related to higher number of retrievable oocytes in the PCOS group [38]. In our study, women with PCOS were evidently characterized by lower Cu concentrations when compared to control group, but when we divided women with PCOS into subgroups according to value of HOMA-IR, we found significantly higher concentrations of Cu in serum of women with HOMA-IR ≥2.0 than in the group of women with HOMA-IR <2.0, while being overweight or obese, established according to BMI value, did not influence Cu concentrations.

The results of previous studies showed that many disorders observed during PCOS such as impaired glucose regulation, type 2 diabetes [16], obesity [15], and inflammation [17] could be associated with the rs2070424 polymorphism of SOD1.

To our knowledge, until now the *rs2070424* polymorphism of *SOD1* in the course of PCOS was not evaluated. Interestingly, we found mainly variant *AA* (93.3%) in the PCOS women and only 4 (6.7%) patients with genotype *AG*, while there are not any patients with *GG* genotype. A study conducted by Silig et al. [14] revealed that the frequencies of *AA*, AG and *GG* genotypes in a healthy Turkish population were found to be % 86.2, % 13.4 and % 0.4 respectively. It is possible that due to a small number of patients, we did not observe any cases of *GG* genotype.

On the other hand, a higher frequency of *AA* genotype could be associated with many disorders. Study conducted by Yin et al. [16] in the Chinese Han population demonstrated that allele A of the *SOD1 rs2070424* polymorphism was more frequent in patients newly diagnosed with impaired glucose regulation and type 2 diabetes than in control group. Moreover, Hernández-Guerrero et al. [15] reported that genotype *GA + GG* of *SOD1* in Mexican women was characterized by higher activity of SOD1 and lower in genotype AA. In addition, similar results were obtained by Spisak et al. in a Polish population, in which authors evaluated the several possible polymorphisms and their association with the risk of Alzheimer’s disease [39]. They claimed that only rs2070424 was significantly related to the risk of Alzheimer’s disease as well as genotype AG and GG were indicated as protective factors for Alzheimer’s disease development [39]. Furthermore, the G allele of the *SOD1 rs2070424* polymorphism decreases the risk of ulcerative colitis; thus, it might be assumed that the *G* allele has a protective role also in this disease [40]. It is possible that lower activity of SOD1 could be associated with the genotype of *AA*, which could be significant in the course of PCOS; therefore, research should be continued.

## 5. Conclusions

1. Women with PCOS are characterized by lower activity of SOD1 and decreased concentrations of Zn and Cu.
2. Higher values of HOMA-IR in the women with PCOS is associated with further drop in SOD1 activity.
3. Possibly, the genotype of AA of the SOD1 rs2070424 polymorphism could be related to lower activity of SOD1, but this part of the study should be continued.

## Figures and Tables

**Table 1:** Characteristics of women with and without PCOS.

Parameters	PCOS; *n* = 60	Control Group; *n* = 15	*p* Value
Age [years]	24.0 (21.0; 29.0)	26.0 (22.0; 39.0)	NS
BMI [kg/m^2^]	22.7 (19.7; 25.1)	23.7 (21.1; 25.4)	NS
WHR	0.8 (0.76; 0.85)	0.8 (0.76; 0.82)	NS
Fasting glucose [mg/dL]	88.0 (84.0; 90.0)	84.1 (81.0; 88.9)	NS
Glucose after OGTT [mg/dL]	98.5 (86.0; 115.0)	not assayed	N/A
Fasting insulin [mU/mL]	6.0 (3.6; 8.9)	6.4 (5.2; 9.0)	NS
Insulin after OGTT [mU/mL]	27.4 (17.8; 47.7)	not assayed	N/A
HOMA-IR	1.3 (0.8; 1.9)	1.3 (1.1; 2.0)	NS
Total cholesterol (mg/dL)	167.5 (148.0; 190.0)	179.0 (167.0; 207.0)	NS
LDL-C (mg/dL)	92.0 (78.0; 113.0)	107.0 (84.0; 127.0)	NS
HDL-C (mg/dL)	55.0 (46.0; 65.0)	58.0 (54.0; 74.0)	NS
Triglycerides (mg/dL)	73.5 (59.5; 118.0)	84.0 (62.0; 122.0)	NS
SOD1 activity [U/L]	5.1 (4.5; 6.0)	9.1 (5.5; 9.9)	<0.001
Zn [µg/L]	857.0 (755.6; 931.5)	946.3 (877.7; 1066.3)	<0.001
Cu [µg/L]	848.6 (746.9; 1009.8)	1195.2 (1164.1; 1336.4)	<0.001
Cu/Zn ratio	1.0 (0.9; 1.1)	1.3 (1.1; 1.4)	<0.001

Legend: Values shown as median (1st quartile, 3rd quartile); PCOS—polycystic ovary syndrome; BMI—body mass index, WHR—waist-to-hip ratio, OGTT—oral glucose tolerance test; HOMA-IR—homeostatic model assessment for insulin resistance; LDL-C—low-density lipoprotein; HDL-C—high-density lipoprotein; SOD—superoxide dismutase; Zn—zinc; Cu—copper; N/A—not applicable; NS—not significant.

**Table 2:** Comparison of analyzed parameters in the group of women with PCOS divided according to the value of HOMA-IR or BMI.

Parameters	PCOS		*p* Value
	HOMA-IR < 2.0; *n* = 43	HOMA-IR ≥ 2.0; *n* = 17	
SOD activity [U/L]	5.2 (4.7; 6.3)	4.7 (4.3; 5.5)	0.046
Zn [µg/L]	858.7 (769.7; 942.8)	818.3 (752.7; 920.7)	NS
Cu [µg/L]	813.8 (724.1; 989.0)	958.1 (832.1; 1077.2)	0.012
Cu/Zn ratio	1.0 (0.9; 1.1)	1.1 (1.0; 1.2)	0.015
LH [lU/L]	5.8 (3.4; 9.1)	8.6 (5.6; 12.5)	NS
FSH [lU/L]	5.8 (5.0; 7.0)	5.9 (5.2; 6.5)	NS
FT4 [ng/dL]	1.2 (1.0; 1.3)	1.2 (1.0; 1.3)	NS
TSH [mU/L]	1.9 (1.4; 2.9)	1.9 (1.3; 2.5)	NS
SHBG [nmol/L]	70.0 (46.7; 86.6)	43.1 (22.8; 59.0)	0.004
Total testosterone [ng/mL]	0.4 (0.2; 0.4)	0.5 (0.4; 0.6)	0.016
Free testosterone [pg/mL]	1.6 (1.1; 2.4)	2.3 (1.2; 3.3)	NS
Androstenedione [ng/mL]	2.5 (1.7; 3.5)	4.0 (2.6; 4.8)	0.024
17-β-E2 [pg/mL]	30.5 (21.5; 48.6)	37.8 (30.2; 50.5)	NS
17-OHP [nmol/L]	1.7 (1.1; 1.9)	2.1 (1.6; 2.4)	0.037
	BMI < 25.0; *n* = 40	BMI ≥ 25.0; *n* = 20	
SOD activity [U/L]	5.6 (4.3; 6.5)	5.1 (4.5; 5.3)	NS
Zn [µg/L]	867.6 (802.9; 942.8)	834.2 (733.1; 959.1)	NS
Cu [µg/L]	859.2 (742.6; 1003.9)	852.3 (772.2; 1042.1)	NS
Cu/Zn ratio	1.0 (0.9; 1.1)	1.0 (0.9; 1.3)	NS
LH [lU/L]	5.9 (22.2; 50.4)	7.8 (3.4; 13.0)	NS
FSH [lU/L]	6.4 (5.0; 7.1)	6.1 (5.1; 6.6)	NS
FT4 [ng/dL]	1.2 (1.1; 1.4)	1.1 (1.0; 1.2)	NS
TSH [mU/L]	1.8 (1.5; 2.5)	1.7 (1.2; 2.5)	NS
SHBG [nmol/L]	76.3 (52.7; 93.4)	26.7 (17.1; 44.5)	0.001
Total testosterone [ng/mL]	0.3 (0.2; 0.4)	0.5 (0.4; 0.7)	0.001
Free testosterone [pg/mL]	1.6 (1.0; 2.2)	2.5 (1.3; 3.7)	0.005
Androstenedione [ng/mL]	2.6 (1.7; 3.5)	4.0 (1.8; 4.9)	0.038
17-β-E2 [pg/mL]	34.4 (22.2; 50.4)	36.6 (28.6; 51.8)	NS
17-OHP [nmol/L]	1.6 (1.0; 2.0)	2.3 (1.6; 2.5)	0.030

Legend: Values shown as median (1st quartile, 3rd quartile); PCOS—polycystic ovary syndrome; SOD—superoxide dismutase; Zn—zinc; Cu—copper; LH—luteinizing hormone; FSH—follicle-stimulating hormone; FT4—free thyroxine; TSH—thyrotropin; SHBG—sex hormone-binding globulin; 17-β-E2—17-β-estradiol; 17-OHP—17-α-hydroxyprogesterone; NS—not significant.

**Table 3:** The activity of SOD1 and Zn, Cu concentration as well as Zn/Cu ratio in the group of women with PCOS divided according to genotype of rs2070424.

Parameters	*AA* *n* = 56	*AG* *n* = 4
SOD activity [U/L]	5.1 (4.5; 6.3)	5.4 (4.9; 5.9)
Zn [µg/L]	860.6 (770.9; 931.5)	848.5 (666.3; 1022.0)
Cu [µg/L]	856.5 (738.3; 1012.3)	846.4 (824.2; 915.2)
Cu/Zn ratio	1.0 (0.9; 1.1)	1.1 (0.8; 1.4)

Legend: Values shown as median (1st quartile, 3rd quartile); PCOS—polycystic ovary syndrome; SOD—superoxide dismutase; Zn—zinc; Cu—copper; rs2070424 genotypes: AA and AG.

**Table 4:** Statistically significant correlation coefficient in whole group of PCOS women between BMI, WHR, glucose metabolism and lipid profile parameters and selected hormone concentrations with SOD1 activity and Zn and Cu concentrations.

Correlation	SOD1 Activity [U/L]	Cu [µg/L]	Zn [µg/L]
BMI [kg/m^2^]	−0.27; 0.041	0.42; <0.001	NS
WHR	−0.33; 0.020	0.36; 0.005	NS
Fasting glucose [mg/dL]	NS	NS	0.30; 0.010
Fasting insulin [uIU/mL]	NS	0.26; 0.024	NS
Glucose after OGTT [mg/dL]	−0.27; 0.043;	NS	NS
Insulin after OGTT [uIU/mL]	−0.34; 0.011	0.23; 0.049	NS
HOMA-IR	NS	0.28; 0.016	NS
Triglycerides [mg/dL]	−0.36; 0.007	0.36; 0.002	NS
LH [lU/L]	−0.28; 0.041	NS	NS
FSH [lU/L]	NS	NS	NS
FT4 [ng/dL]	−0.30; 0.026	NS	NS
TSH [mU/L]	−0.33; 0.010	NS	NS
SHBG [nmol/L]	NS	−0.30; 0.011	NS
Total testosterone [ng/mL]	NS	NS	NS
Free testosterone [pg/mL]	NS	NS	NS
Androstenedione [ng/mL]	NS	NS	NS
17-β-E2 [pg/mL]	NS	NS	NS
17-OHP [nmol/L]	NS	NS	NS

Legend: Values shown as median (1st quartile, 3rd quartile); PCOS—polycystic ovary syndrome; SOD—superoxide dismutase; Zn—zinc; Cu—copper; LH—luteinizing hormone; FSH—follicle-stimulating hormone; FT4—free thyroxine; TSH—thyrotropin; SHBG—sex hormone-binding globulin; 17-β-E2—17-β -estradiol; 17-OHP—17-α-hydroxyprogesterone; NS—not significant.

## Data Availability

All relevant data in the current study are available from the corresponding author on request.
